# Rapid digital light 3D printing enabled by a soft and deformable hydrogel separation interface

**DOI:** 10.1038/s41467-021-26386-6

**Published:** 2021-10-18

**Authors:** Jingjun Wu, Jing Guo, Changhong Linghu, Yahui Lu, Jizhou Song, Tao Xie, Qian Zhao

**Affiliations:** 1 Ningbo Research Institute Zhejiang University, Ningbo, 315807 China; 2 State Key Laboratory of Chemical Engineering, College of Chemical and Biological Engineering, Zhejiang University, Hangzhou, 310027 China; 3 ZJU-Hangzhou Global Scientific and Technological Innovation Center, Hangzhou, 311215 China; 4 Department of Engineering Mechanics, Soft Matter Research Center, and Key Laboratory of Soft Machines and Smart Devices of Zhejiang Province, Zhejiang University, Hangzhou, 310027 China

**Keywords:** Polymers, Polymers, Design, synthesis and processing

## Abstract

The low productivity of typical 3D printing is a major hurdle for its utilization in large-scale manufacturing. Innovative techniques have been developed to break the limitation of printing speed, however, sophisticated facilities or costly consumables are required, which still substantially restricts the economic efficiency. Here we report that a common stereolithographic 3D printing facility can achieve a very high printing speed (400 mm/h) using a green and inexpensive hydrogel as a separation interface against the cured part. In sharp contrast to other techniques, the unique separation mechanism relies on the large recoverable deformation along the thickness direction of the hydrogel interface during the layer-wise printing. The hydrogel needs to be extraordinarily soft and unusually thick to remarkably reduce the adhesion force which is a key factor for achieving rapid 3D printing. This technique shows excellent printing stability even for fabricating large continuous solid structures, which is extremely challenging for other rapid 3D printing techniques. The printing process is highly robust for fabricating diversified materials with various functions. With the advantages mentioned above, the presented technique is believed to make a large impact on large-scale manufacturing.

## Abstract (web summary)

The commercial application of 3D printing is limited by time constraints and current efforts to speed the process are economically inefficient. Here the authors report on the use of a green and inexpensive hydrogel to separate the interface and cured components in common stereolithographic processes to allow for rapid printing.

## Introduction

3D printing is a layer-by-layer additive manufacturing technique, which enables the fabrication of customized or complex structures without the costly and time-consuming mold-making procedure^1–4^. However, current 3D printing for polymers is mainly limited to prototyping rather than fabricating final products. Its market share is only 7‰ in comparison with traditional liquid molding^5^. A major reason for this is that the low productivity originated from the layer-by-layer process is uneconomic for large-scale manufacture. How to substantially increase the productivity in an economically feasible fashion is critical to the further development of this booming technique^6^. In comparison with point-by-point 3D printing techniques such as fused deposition modeling and stereolithography, digital light processing (DLP) 3D printing exhibits significant advantages in printing speed due to its layer-by-layer processing feature^7–9^. Therefore, DLP is considered as a most promising 3D printing technique that might make an enormous change towards large-scale manufacture if a further striding improvement of the productivity can be realized.

DLP 3D printing can be divided into a top-down form and a bottom-up form according to the moving direction of the building platform. The latter one is currently more popular since it stands out with several prominent advantages such as better vertical resolution, less resin consumption, higher leveling rate of liquid resins, and more diversified adaptability of materials^10,11^. A separation interface between the resin and the curing window is required to avoid bonding of the two parts. Fluorinated ethylene propylene (FEP) membrane has become a commercial solution due to its low surface energy nature, but the adhesive force is still too large especially for printing continuous solid structures^12^. To address this problem, innovative releasing materials or techniques have been invented to achieve rapid 3D printing. These techniques can generally be classified into two methods, namely, polymerization inhibition and slippery boundary. Continuous liquid interface printing (CLIP) was innovated, which uses polymerization inhibition to create a reaction “dead layer” enabled by oxygen permeable membrane^13,14^ or volumetric photoinhibition patterning^15^. This dead layer prevents adhesion between the emerging part and the bottom of the print vat. Flowing fluorinated oil bed^16^ and fluorinated oil infused polydimethylsiloxane (PDMS)^17^ were developed to provide a solid-liquid slippery boundary which inhibits the direct contact between the cured resin and the solid interface. These elegant methods, however, require sophisticated facilities (e.g., additional photo exposure source and flowing oil bed) or much more costly consumables than FEP (e.g., oxygen permeable membrane and fluorinated oil infused PDMS), which are extremely hard to be commercialized. A simple, economic, and universal release technique is still highly expected to bring 3D printing into a substantially greater stage towards large-scale manufacture.

Hydrogels are crosslinked polymer networks swollen by water with content typically higher than 90%^18–23^. As mostly aqueous materials, hydrogels have shown promises in various bio-related fields due to their high water content and softness which provides excellent compatibility with biological tissues^24,25^. These two characteristics, however, limit their potential for wider applications in particular for non-bio-related manufacturing.

In this work, we present that an extraordinarily soft hydrogel can be utilized as an excellent separation interface to enable rapid printing. In contrast to the polymerization inhibition and slippery boundary strategies, the hydrogel interface can undergo large recoverable deformation along the thickness direction to gradually reduce the adhesion force which is a distinct separation mechanism. Except for the application of the hydrogel interface, the printing process and the facility has no difference with a common technique whereas the printing speed increases by an order of magnitude.

## Results

### Setup, process, and hydrogels

**Fig. 1:**
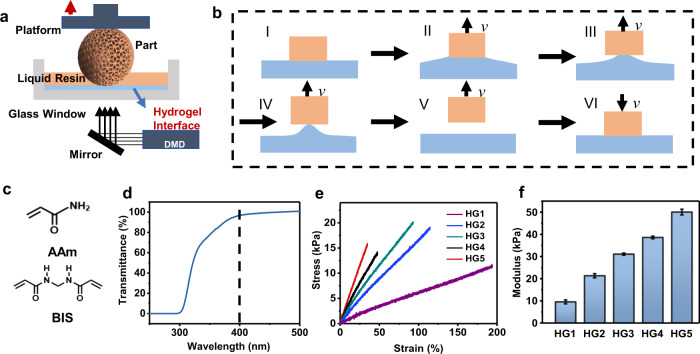
Setup, process, and hydrogels. **a** Schematic demonstration of the DLP printing process equipped with a hydrogel layer as the separation interface between the glass window and the liquid resin. **b** Schematic process of the peeling-based separation on a soft and deformable interface. **c** Chemical structures of the monomers for the hydrogel. **d** Transparency of the curing window composed of the glass tank and the hydrogel layer. **e** Tensile stress-strain curves of the hydrogels with different compositions. **f** Tensile modulus of different hydrogels. Error bars: Standard Deviation (*n* = 3).

The setup of the 3D printing system is schematically illustrated in Fig. 1a. The process of DLP printing consists of three actions, namely, photocuring of liquid resins, movement of the build platform, and the refilling of liquid resin. While the photocuring time of many commercial precursors can be reduced to one second per layer or even less, the movement and leveling actions are usually more time-consuming by an order of magnitude. The slow separation between the newly cured layer and the curing window of the resin tank is required to relax the adhesive force of the two parts. Otherwise, cohesive failure of the cured objects or the curing window will occur. The separation force mainly originates from the molecular adhesion between the cured part and the separation interface. While most of the reported work focuses directly on lowering this adhesion, we hypothesize that a soft and deformable interface could result in a peeling-based separation process to gradually release the adhesion force (Fig. 1b). To confirm this, soft hydrogels as the interfaces were synthesized in-situ on the glassy curing window via aqueous radical copolymerization of acrylamide (AAm) as the main monomer and *N*,*N*′-methylenebis(acrylamide) (BIS) as the crosslinker (Fig. 1c). The hydrogel provided excellent transparency (>95%) for the UV curing light with a central wavelength of 405 nm (Fig. 1d), thus will not hinder the photocuring of the resin. The achievable smallest feature size is approximately 100 μm (Supplementary Fig. 1) which coincides with the physical pixel size of the light source. Thus, the introduction of the hydrogel interface does not noticeably affect the intensity and the propagation of the light source. Mechanical properties of the hydrogels were tuned via the crosslinker (BIS) content. The weight percentage of BIS to AAm is 1 wt%, 2 wt%, 3 wt%, 4 wt%, and 5 wt% for the hydrogels labeled as HG1, HG2, HG3, HG4, and HG5, respectively (Fig. 1e, f). In comparison with FEP film with a modulus of 400 MPa (Supplementary Fig. 2), the modulus of the hydrogels varies from 10 kPa to 50 kPa. Among the hydrogels, the HG1 sample exhibits the lowest modulus and the highest deformability, which should be the best candidate according to our hypothesis. Further decrease of the modulus would lead to sticky hydrogels due to the inadequate crosslinking.

The hydrogels are immiscible with most of common photosensitive resins, which is the basis of the separation interface. Taking 1,6-hexanediol diacrylate (HDDA) for instance, the resin loaded in the hydrogel-integrated tank for one month can still be printed without any problem, implying no critical diffusion of the resin across the interface. A real-time separation process using an HG1 hydrogel with a 4 mm thickness (labeled as HG1-4) was recorded when printing a cylinder with a 20 mm diameter (Supplementary Fig. 3, Supplementary Movie 1). Upon movement of the printing platform, the detachment initiates at the circumference of the contact area between the cured sample and the hydrogel interface and propagates inwards to the center, exhibiting a stepwise peeling due to the soft nature of the hydrogel interface. Simultaneously, the liquid resin is refilled into the gap to be cured by the next exposure pattern. Such a series of actions took less than 0.2 s due to the good and rapid resilience of the hydrogel. Such a separation process conforms to our hypothesis. Although the hydrogels are immiscible with the liquid resin, the contact angle of HDDA on the hydrogel is approximately 35° (Supplementary Fig. 4). It implies that the interface does not provide a low surface energy nature, thus the mechanism is distinct from the slippery boundary strategies^17^.

### Evaluation of the separation force and the printability

**Fig. 2:**
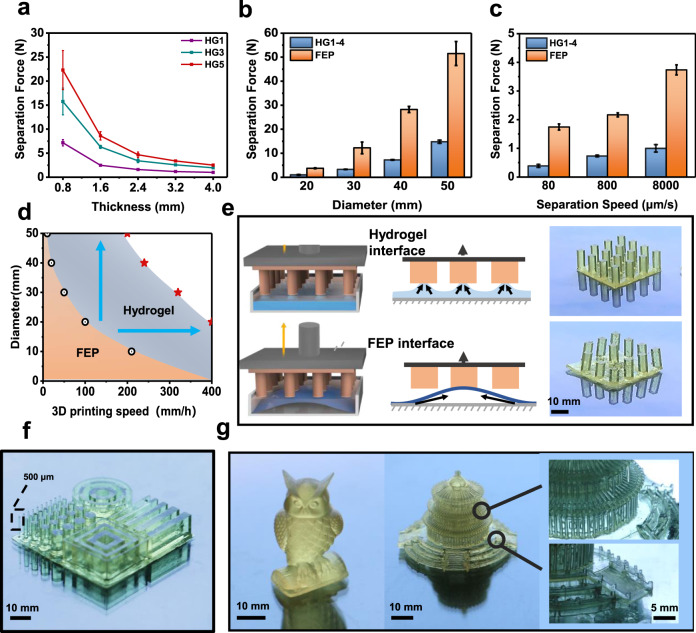
Evaluation of the separation force and the printability. **a** Separation force when printing a cylinder of 20 mm diameter using hydrogels with various modulus and thickness. **b** Separation force when printing cylinders of various diameters using the HG1-4 hydrogel. **c** Separation force upon various separation speeds. **d** Achievable productivity when printing cylinders of various diameters using FEP and the hydrogel interface. The back circles and the red stars represent the experimental results using the FEP and hydrogel interfaces respectively. **e** Scheme of different separation mechanism of FEP and the hydrogel interface when printing discrete object array. **f** Photograph of a customized testing model printed at a speed of 400 mm/h. **g** Photographs of the printed owl (400 mm/h) and the Temple of Heaven (200 mm/h). Error bars: Standard Deviation (*n* = 3).

Real-time separation force was measured via mounting a force sensor onto the platform of the 3D printer (Supplementary Fig. 5). A cylinder model with a 20 mm diameter was printed via photocuring of the HDDA resin. Maximum separation forces were recorded when hydrogels with various modulus and thickness were applied (Fig. 2a). As expected, hydrogels with lower modulus provide a lower separation force. At a same hydrogel thickness of 0.8 mm, the force is 7.5 N for HG1, which is approximately one third of that for HG5. On the other hand, it is intriguing that the separation force reduces dramatically with the increase of thickness of the hydrogel interfaces. The force using a 4 mm HG1 is only 1 N, which is an order-of-magnitude smaller than that using a 0.8 mm HG1. The influence of both the modulus and the thickness on the separation force is in accord with our hypothesis. It is obvious that softer materials provide smaller separation force with the same interfacial interaction properties. Whereas for the effect of thickness, the realistic strain and the corresponding separation force for a thicker interface will definitely be smaller when a certain apparent displacement along the thickness direction takes place. From another point of view, as the modulus of the hydrogel gets smaller or the thickness becomes larger, the stress will be more concentrated near the edge of the printed sample^26,27^. Consequently, a smaller load is required to drive the edge crack to propagate, which leads to a smaller separation force. Further increase of the thickness would approach nearly a plateau toward a semi-infinite interface in reducing the separation force and a decrease of the transparency of the hydrogel which does not benefit the photopolymerization (Supplementary Fig. 6).

As such, HG1-4 was chosen to act as the separation interface in the following study. The separation force increased exponentially with the exposure area^28^. It is therefore a great challenge to print large continuous solid structures. The separation force of printed cylinder models with various diameters is presented in Fig. 2b. The force is significantly smaller using the hydrogel than an FEP interface. When printing a cylinder with a diameter of 50 mm, the hydrogel interface presents a 13 N force which is one fourth of that using FEP. The lifting velocity of the printing platform is also a critical parameter that will impact both the separation force and the actual printing speed. Although a larger lifting velocity can accelerate the printing speed, it will also lead to a larger separation force due to the viscoelasticity of the interface^29,30^. As shown in Fig. 2c, even at a relatively large velocity of 8000 μm/s (the maximum lifting velocity of the current printer), the separation force is 1 N when printing a 20 mm cylinder when the hydrogel interface is applied, which is one third of that using FEP.

The low separation force of the hydrogel interface is beneficial for achieving a high printing speed. The exposure time for a 0.1 mm layer is fixed at 0.5 seconds. The lifting velocity of the platform is set at 8000 μm/s, and the lifting distance of the platform varies from 0.5 mm to 5 mm according to the solid cross-sectional area (Supplementary Table 1). As shown in Fig. 2d, an empirical maximum printing speed for a specific geometry (cylinders with different diameters) can be obtained under the limitation of the current commercial hardware. A printing speed of 400 mm/h is achievable when printing cylinders with diameters less than 20 mm (Supplementary Movies 2 and 3). When printing cylinders with a larger diameter, the larger separation force leads to a greater deformation of the hydrogel interface. The lifting distance of the platform from the interface shoud correspondingly increase to guarantee the replenishment of the liquid resin. Therefore, it takes longer to separate from the interface at the same separation speed (Supplementary Table 1). As a result, the printing speed needs to be lowered to 200 mm/h for a cylinder with a diameter of 50 mm (Supplementary Fig. 7). Notably, fabricating large continuous solid structures like this is extremely difficult for other bottom-up DLP 3D printing methods due to the large separation force. An insufficient lifting distance and an excessive lifting velocity will lead to local defects or even total sticking of the objects to the FEP (Supplementary Fig. 8). When FEP is applied, the separation force is generally larger than that for the hydrogel interface as shown in Fig. 2b, c. Thus, the reliable printing speed using FEP is much smaller. When printing large continuous solid structures (e.g. cylinder with 50 mm diameter), the speed is only approximately 10 mm/h, which is twentieth of that using HG1-4. To print a single cylinder with a 10 mm diameter in contrast, the speed can reach 200 mm/h. Although fast, the print process is hypercritical for diversified geometries. For instance, when printing a 4 × 4 cubic array with a cross-sectional dimension of 5 mm × 5 mm, the parts become defective at a speed of 200 mm/h (Fig. 2e). In contrast, the printing is highly reliable when the hydrogel interface is applied. The same array model can be accurately prepared at a printing speed of 400 mm/h. The difference in the printing reliability is originated from distinct separation mechanisms of the two interfaces. As schematically illustrated in Fig. 2e, the hydrogel interface can be locally dragged along the thickness direction thanks to its ultra-low modulus and the fixed boundary condition at the bottom. This ensures that the separation process of discrete patterns would not affect each other. In contrast, the FEP membrane is only clamped at the periphery, which allows free deformation of the whole membrane when being dragged. This global peeling mode results in interdependence of the discrete patterns. Specifically, the deformations of the neighboring pillars on the FEP membrane are transmitted through the membrane force and the rigidity of the membrane further aggravates this problem. The peripheral part of the FEP membrane is stretched more severely than the center due to the circumferential confinements. This would bend the arrayed pillars and thus impair the printing accuracy. In this case, the actual speed of commercial FEP-equipped printing technologies is commonly lower than 40 mm/h, which is an order-of-magnitude smaller than that using the hydrogel interface.

The rapid printing does not sacrifice the printing resolution (Fig. 2f, g). A customized testing model shows that high-aspect-ratio cylinders with 500 μm diameter can be steadily fabricated at a printing speed of 400 mm/h. A cyclic tensile test of the hydrogel was conducted. The modulus and resilience exhibit no obvious decline after ten thousand tensile cycles, indicating good mechanical stability (Supplementary Fig. 9). The real-time separation force during ten thousand consecutive separations was also recorded. The force values show no obvious fluctuation, indicating good compatibility between the printing resin and the hydrogel interface without unfavorable resin diffusion (Supplementary Fig. 10). Also, the hydrogel interface preserves good transparency after ten thousand separations (Supplementary Fig. 11). In this case, the printing process is robust withstanding thousands of consecutive separations.

Managing heat dissipation is challenging for rapid printing technologies. For the current system, the hydrogel interface is advantageous due to the high specific heat of water. This is proven by real-time temperature monitoring using infrared camera during printing. As shown in Supplementary Fig. 12, the temperature of the cured part reached a maximum value of approximately 75 °C after printing for 2 min, whereas that of the immediate contract hydrogel area was around 50 °C. A one-week consecutive printing test was conducted to examine the durability of the hydrogel interface. The morphology of the printed objects did not show any detectable variation. Notably, the weight of the hydrogel did not change since the top liquid resin prevented water evaporation.

### Comparison of the separation mechanisms

At first glance the separation mechanism using the hydrogel is similar to that using PDMS which was once popular as an interface material in the early period of DLP 3D printing. PDMS happens to provide three characters those can reduce the separation force, that is, deformability, slipperiness, and curing inhibition. However, the separation force using PDMS is still too large to realize rapid printing, which has been proved in previous studies^17^. To achieve rapid printing, the characters and the enabling mechanism need to be pushed to extreme ends. Two good examples are oxygen permeable membrane enabling very strong curing inhibition^13^ and fluorinated oil infused PDMS enabling excellent slipperiness^17^. In comparison, we are here pushing the deformability to an extreme using an ultra-soft hydrogel. To discover such a mechanism and the enabling material is nontrivial. First, the optimized hydrogel provided Young’s modulus as low as 10 kPa, which is very challenging for other soft materials including PDMS. Even for hydrogels, 10 kPa is an unusually low value, which is desired in few applications. Hydrogels with a larger modulus (e.g., 50 kPa, which are already a very small value) cannot achieve a rapid printing, since the corresponding adhesion force is similar to that using FEP film. Second, the thickness of the interface (4 mm) is an unusually large value for an interface, which would be aware by few researchers.

**Fig. 3:**
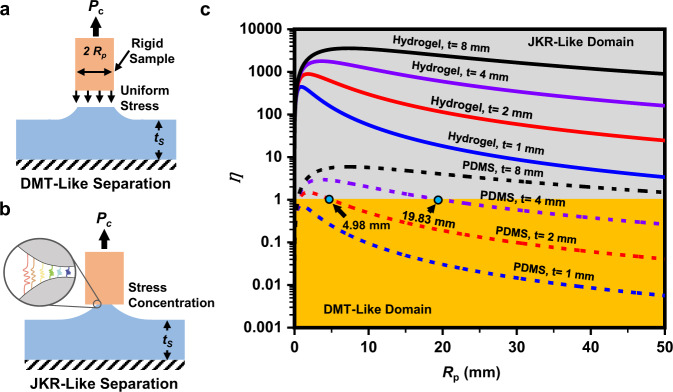
Comparison of the separation mechanisms using PDMS and hydrogel interfacial layers. **a** Illustration of the DMT-like separation. **b** Illustration of the JKR-like separation. **c** JKR-DMT transition phase diagram of the separation process for PDMS and hydrogel interfaces. *P*_c_ is the separation force, *R*_P_ is the radius of the punch, *t*_s_ is the elastic modulus of the hydrogel.

The mechanistic difference of PDMS and the hydrogel during separation is further illustrated in Fig. 3. Under limited thickness *t*_*s*_ and a large printed diameter *R*_*p*_, the separation process between the printed sample and PDMS interface is strength-limited (namely DMT-like)^31–33^. In this case, the interfacial stress is uniform and the sample is separated suddenly as a whole (as illustrated in Fig. 3a). This yields a large separation force due to the adhesion as well as a very high suction force from rapid liquid refilling. For the hydrogel interface, the intrinsic ultra-softness ensures that the interfacial separation proceeds via a crack-propagation (or JKR-like) mechanism. Under this condition, the interfacial stress is concentrated at the periphery, and the separation proceeds gradually from the periphery to the center (as illustrated in Fig. 3b, also referred as the peeling-based separation in Fig. 2b). The gradual release process can reduce both the adhesion force through the stress concentration and the suction force via gradual refilling of the liquid resin. Consequently, the separation force will maintain a very low value. Quantitatively, the JKR to DMT transition is dominated by a non-dimensional factor $\eta =\frac{\pi \left(1-{v}^{2}\right)}{2}\cdot \frac{{{\sigma }_{m}}^{2}\cdot {R}_{p}}{{E}_{S}{G}_{c}\cdot \kappa }$ ^32,33^, where ${\sigma }_{m}$ is the theoretical interfacial adhesion strength, *R*_*p*_ is the radius of the sample, *E*_*s*_ and *v* are the elastic modulus and Poisson’s ratio of the compliant interface, respectively, and $\kappa$ is the normal contact stiffness of the interface. When the parameter $\eta$ is smaller than one, the separation process is strength-limited. The results based on the above analysis were presented in Fig. 3c using typical adhesion and elastic parameters of PDMS and hydrogel. For a given sample diameter and separation film thickness, hydrogel lies safely within the JKR-like domain. For the PDMS, the interfacial separation is mostly DMT-like, unless the film is abnormally thick (>8 mm) and the printed sample diameter is relatively small. Overall, the operation window to achieve easy separation is much wider for the hydrogel, which is critical for robust and fast DLP printing.

### Mechanics modeling and analysis

**Fig. 4:**
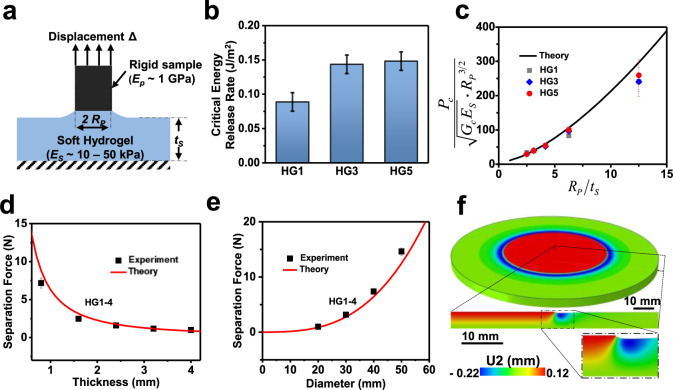
Mechanics modeling and analysis of the printing process equipped with a hydrogel interface. **a** Illustration of the mechanic model. **b** The critical energy release rate for hydrogel/HDDA sample interface. **c** Comparison of the predicted and measured separation forces under different hydrogel thicknesses and compositions. **d** Comparison of the predicted and measured separation forces under various hydrogel thickness. **e** Comparison of the predicted and measured separation forces under various sample diameters. **f** FEA results of the typical deformed shape and vertical displacement distribution of the hydrogel layer (HG1-4, sample diameter: 50 mm). *P*_c_ is the separation force, *G*_c_ is the critical energy release rate of the punch/hydrogel interface, *R*_P_ is the radius of the punch, *E*_s_ and *t*_s_ are the elastic modulus and thickness of the hydrogel, *E*_p_ is the elastic modulus of the cured part. Error bars: Standard Deviation (*n* = 3).

To provide more insights into the underlying mechanics of the hydrogel interface, a linear fracture mechanics model by assuming that the separation force is dominated by the adhesive force is developed. This treatment is reasonable since the suction force caused by the refilling of fluid (i.e., resin) is relatively small when the printed sample is gradually detached from the soft hydrogel. Considering that the cured sample (~1.0 GPa) is much stiffer than the soft hydrogel (10–50 kPa), the separation process can be modeled as a rigid punch detached from a soft adhesive layer fixed at the bottom (Fig. 4a), which gives the separation force in the non-dimensional form^34,35^ for the incompressible hydrogel as

1 $$\frac{{P}_{c}}{\sqrt{{G}_{c}{E}_{S}}\cdot {{R}_{P}}^{\frac{3}{2}}}=4\sqrt{\frac{2\pi }{3}}\cdot \sqrt{1+\frac{4}{3}\frac{{R}_{P}}{{t}_{S}}+\frac{4}{3}{\left(\frac{{R}_{P}}{{t}_{S}}\right)}^{3},}$$

where *P*_c_ is the separation force, *G*_c_ is the critical energy release rate of the punch/hydrogel interface, *R*_P_ is the radius of the punch, *E*_s_ and *t*_s_ are the elastic modulus and thickness of the hydrogel, respectively. The critical energy release rates *G*_c_ of HG1, HG3, HG5 hydrogel/HDDA (cured sample) are 0.08862 J/m^2^, 0.14346 J/m^2^, and 0.14808 J/m^2^ (Fig. 4b), respectively, which are obtained by fitting the corresponding measured separation forces (Fig. 2a) with the hydrogel thickness of 3.2 mm when the separation speed is 8000 μm/s and the punch diameter is 20 mm. One thing that should be noted is that *G*_c_ is peeling-rate-dependent and the separation speed is fixed to experimentally measure the separation force. Equation (1) directly shows that the separation force is proportional to the elastic modulus *E*_s_ of the hydrogel, while a larger hydrogel thickness *t*_s_ will lead a smaller separation force. This further validates our former discussion that softer and thicker hydrogel provide smaller separation force with the same interfacial interaction properties.

Figure 4c shows the predicted thickness-dependent separation forces as compared with experiments. Their good agreement validates the accuracy of the mechanics model, which is further supported by Fig. 4d, e, where both the trend and values of separation force under various hydrogel thickness and sample diameters are captured. As the hydrogel thickness increases, the gain in reducing the separation force through a thicker hydrogel gradually reduces (Figs. 2e and 4d). Such a result can be explained by Eq. (1). When *t*_s_ is small, the term of *R*_p_*/t*_s_ in Eq. (1) is large and its contribution to *P*_c_ is significant. At a high value range of *t*_s_, the value of *R*_p_*/t*_s_ is small and its contribution to *P*_c_ becomes minor, leading to the plateau in separation force.

Notably, the separation force is proportional to the third power of the printed part diameter when the deformable hydrogel interface is applied as shown in Eq. (1). Whereas for other previous fast printing techniques which normally introducing a liquid layer between the cured part and the exposure window (e.g., the CLIP strategy), the separation force is proportional to the fourth power of the diameter ($F=\frac{3\pi * \mu V}{2* {h}^{3}}* {R}^{4}$)^36^. In contrast to this liquid-solid separation, the present hydrogel strategy fundamentally provides advantages when printing the parts with large cross-sections due to the solid-solid separation mode.

Finite element analysis with details given in materials and methods is performed to obtain the separation displacement for the large-diameter HDDA cured sample/hydrogel (HG1) interfaces with a relatively large hydrogel thickness (4 mm) used in our experiment. The deformed shape and the vertical displacement distribution in the hydrogel (diameter: 50 mm) are shown in Fig. 4f. The deformations are concentrated at the interface between the sample and the hydrogel due to the soft nature of the hydrogel, which further confirms that the derofmaiton of the hydrogel interface is localized and will not influence the neighboring smaple in an array printing.

### Resins compatibility

**Fig. 5:**
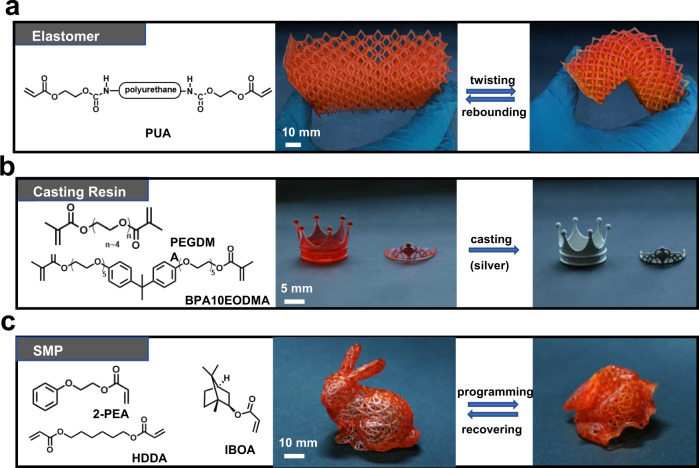
Diversity of the resins that are compatible with the hydrogel interface. **a** Elastomers. **b** Casting resins. **c** Shape memory polymers.

The hydrogel interface exhibits a good printability towards various liquid resins, which is critical for practical applications. Besides the rigid acrylate resin (HDDA) used above, three different resins are printed at a speed of 200 mm/h to obtain objects enabling diversified functions, including a polyurethane acrylate rubber, a castable resin, and a shape memory polymer (SMP). The polyurethane acrylate oligomer is commonly formulated into the resins to improve the mechanical property. The molecular of a general castable resin is rich of ethoxyl groups to guarantee an easy and complete burning out of the printed parts. These two widely used precursors both exhibit certain hydrophilicity. Nevertheless, the printing stability is still excellent. The reason for such may rely on that the polyacrylamide is immiscible with polyethylene glycol in water^37^. The printed elastomeric lattice exhibits a good resilience as shown in Fig. 5a and Supplementary Fig. 13. For the castable resin, the printed rigid parts can perfectly convert into silver ones (Fig. 5b) after being fully burned out confirmed by the thermogravimetric measurement (Supplementary Fig. 14). To go a step further, an acrylate-based SMP is formulated and printed. The shape of the printed SMP rabbit can be arbitrarily programmed and fully recovered across the transition temperature at around 40 °C (Fig. 5c and Supplementary Fig. 15). Direct 3D printing of objects enabling shape memory effect, namely 4D printing, is an emerging strategy to promote device applications that require sophisticated shapeshifting^38–41^.

The PUA-containing resin, the castable resin, and the non-water-soluble acrylate monomers presented in our work have already occupied the majority of the practically applied resins. Having said that, the hydrogel interface does have some limitations on the photocuring resins. Although the castable resin containing water-soluble PEGDMA (40 wt%) (Fig. 5b) presented excellent printing stability, pure PEGDMA cannot be printed using the hydrogel interface because the cured part would suck water from the hydrogel. In addition, the technique is incompatible with water soluble small molecular monomers. However, water-soluble photo-curing precursors are not commonly used in stereolithographic 3D printing for real-world applications due to the poor water-resistance of the cured parts.

## Discussion

In this work, an extraordinarily soft thick hydrogel was directly integrated into a commercial bottom-up DLP printer as a separation interface. The two unusually applied properties enable large deformation along the thickness direction, which significantly reduces the separation force during the consecutive layer-by-layer printing process. This technology shows the advantages of high printing speed, good resin compatibility, and outstanding printing reliability for diversified geometries such as large cross-sectional areas and discrete arrays. According to the unique separation mechanism, constructing surface structure (e.g., pixelated surface) may further decrease the separation force, which would be investigated in the future. It should be also noted that this work aims to develop a 3D printing technique with well-balanced attributes including cost, robustness, and productivity, instead of achieving a record printing speed. This is critical for large-scale manufacturing. Specifically, our vertical printing speed is approximately half of the record speed^13,15^, but it shows an exceptional printing stability. In addition, our technique can be readily integrated with commercial low cost DLP printers without the need for any hardware upgrade. It is expected that the hydrogel interface can be practically applied in large-scale commercialization.

## Methods

### Materials

Acrylamide (AAm), *N,N*′-methylenebisacrylamide (BIS), 2-phenoxy ethyl acrylate (2-PEA), isobornyl acrylate (IBOA) were purchased from J&K company. 2-hydroxy-4′-(2-hydroxyethoxy)-2-methylpropiophenone (Irgacure 2959), Sudan III were obtained from Aladdin reagent company. Phenylbis(2,4,6-trimethylbenzoyl)phosphine (photo-initiator Irgacure 819) was obtained from TCI. 1,6-Hexanediol diacrylate (HDDA) was offered by Sartomer (Guangzhou) Chemicals Ltd. Ethoxylated bisphenol-A dimethacrylate (EM3265), polyethylene glycol (200) dimethacrylate (EM324) were offered by Eternal Materials Co., LTD. All chemicals were used as received.

### Preparation of hydrogel interface

The monomer AAm and the crosslinker BIS were dissolved in distilled water to form a 90 wt% solution. The photoinitiator Irgacure 2959 was added at a fixed amount of 0.2 wt% of the mixture of AAm and BIS. The weight percentage of BIS to AAm is 1 wt%, 2 wt%, 3 wt%, 4 wt%, and 5 wt% for the hydrogels labeled as HG1, HG2, HG3, HG4, and HG5, respectively. The precursor solution was cured in situ on the glassy resin tank of the 3D printer in a UV chamber (66 mW/cm^2^) for 5 mins. To obtain the expected thickness of the hydrogel interface, the amount of the precursor solution was calculated before it was poured into the tank.

### Transparency measurement

Transparency of the hydrogels was measured using a UV–vis spectrophotometer (Shimadzu UV-2600, Japan). Thickness of the hydrogels ranged from 0.8 mm to 5.6 mm.

### Mechanical tests of the hydrogel

The tensile tests were performed on a universal tensile testing machine (Instron 5944 testing system, USA). The samples were cut into dumbbell shapes with 1 mm thickness. The stretching rate was set at 5 mm/min. The modulus of the hydrogel was caculated as the slope of the stress-strain curve in the strain range of 5–10%. To test the durability of the hydrogel during printing, consecutive stretching-releasing was conducted. A dumbbell shaped HG1 sample was stretched using a fatigue testing machine (CARE measurement & control IPBF-300, China). The sample was kept in a water cell during the test to avoid the dehydration. The stretching rate was set at 60 mm/min.

### Preparation of printing resins

The resin for basic testing of the hydrogel interface is composed of 97 wt% HDDA and 3 wt% photo initiator Irgacure 819. The elastomer resin consists of 75 wt% self-made polyurethane acrylate oligomer (Polytetramethylene ether glycol : Hexamethylene Diisocyanate : 2-(Tert-butylamino)ethyl methacrylate = 1:2:2) and 25 wt% 2-ethylhexyl acrylate. The casting resin consists of 60 wt% EM3265 and 40 wt% EM324. The SMP resin consists of 2 wt% HDDA, 32 wt% 2-PEA, and 66 wt% IBOA. 0.03 wt% Sudan III and 3 wt% I819 were incorporated in the later three resins.

### 3D printing and measurement of the separation force

3D printing was conducted in a commercialized DLP printer mounted with a load cell (SHINING 3D Inc., China). The light intensity is 5.0 mW/cm^2^. To test the separation force, circular light patterns with different diameters were projected. Each layer (100 μm) was illuminated for 2 seconds. The platform lifting speed ranged from 80 μm/s to 8000 μm/s, and the lifting distance varied from 0.5 mm (for diameter less than 20 mm) to 5 mm (for diameter 50 mm).

To test the maximum printing speed for hydrogel interface, the lifting velocity was fixed at 8000 μm/s, and the lifting distance of the platform varied from 0.5 mm (for diameter less than 20 mm) to 5 mm (for diameter 50 mm). For FEP, the lifting velocity was tuned from 80 μm/s to 8000 μm/s, and the lifting distance of the platform varied from 0.5 mm (for diameter less than 20 mm) to 5 mm (for diameter 50 mm). The exposure time for the initial 5 layers was 10 s, and the exposure time was 0.5 s for the rest layers.

### Mechanical analysis

An axisymmetric finite element model is established in ABAQUS to obtain the separation displacement required to detach the cylindrical sample (Young’s modulus: 1.0 GPa, Poisson’s ratio: 0.40) from the hydrogel substrate (HG1-4, Young’s modulus: 10 kPa, Poisson’s ration 0.4999). A perfect bonding is assumed at the sample/hydrogel interface. The hydrogel substrate with its bottom surface fixed has an outer diameter of 100 mm and a thickness of 4 mm. A vertical displacement is applied on the top surface of sample. CAX3H elements are used to discretize the geometry. To obtain Fig. 3c, the theoretical interfacial adhesion strength for PDMS and hydrogel interfaces are assumed to be identical at 35 kPa due to their common root in van der Waals interactions^32^. *E*_s_ are 2 MPa (PDMS) and 10 kPa (hydrogels). The values of *G*_c_ are 0.3 J/m^2^ (PDMS, obtained from literature^33^) and 0.1 J/m^2^ (hydrogel, obtained via experiments in this work).

## Supplementary information

Supplementary Information

Description of Additional Supplementary Files

Supplementary Movie 1

Supplementary Movie 2

Supplementary Movie 3

## Data Availability

The raw/processed data required to reproduce these findings can be made available upon request to corresponding authors.
