# Supplementary material for: Rapid digital light 3D printing enabled by a soft and deformable hydrogel separation interface
Source: Nat Commun. 2021 Oct 18;12:6070. doi: 10.1038/s41467-021-26386-6 (PMC8523520; doi:10.1038/s41467-021-26386-6)
Supplement: Supplementary file 1 — Supplementary Information [file 41467_2021_26386_MOESM1_ESM.pdf]

Supplemental Information

Rapid digital light 3D printing enabled by a soft and deformable hydrogel separation interface

Jingjun Wu[1,2,†], Jing Guo[2,3,†], Changhong Linghu[4†], Yahui Lu[2], Jizhou Song[4,*], Tao Xie[1,2,3], Qian Zhao[1,2,3,*]

[1]Ningbo Research Institute Zhejiang University, Ningbo 315807, China
[2]State Key Laboratory of Chemical Engineering, College of Chemical and Biological Engineering, Zhejiang University, Hangzhou 310027, China.
[3]ZJU-Hangzhou Global Scientific and Technological Innovation Center, Hangzhou 311215, China.
[4]Department of Engineering Mechanics, Soft Matter Research Center, and Key Laboratory of Soft Machines and Smart Devices of Zhejiang Province, Zhejiang University, Hangzhou 310027, China.
† These authors contributed equally to this work.
*Correspondence: qianzhao@zju.edu.cn and jzsong@zju.edu.cn

This PDF file includes:
Supplementary Table 1.
Supplementary Figure 1 to 15.

Other Supplementary Materials for this manuscript include the following:
Supplementary Movie 1 to 3

Supplementary Table 1. Printing parameters and printing speed for cylinders with different diameters.

Cylinder diameter (printing speed)	Time for the separation of the printed part and interface*	Lifting distance of the platform during 3D printing**
20 mm (400 mm/h)	0.2 s	0.5 mm
30 mm (320 mm/h)	0.26 s	1 mm
40 mm (240 mm/h)	0.35 s	3 mm
50 mm (200 mm/h)	0.48 s	5 mm

*Data captured from the real-time separation process during printing.
**The lifting distance is determined according to the printing result.

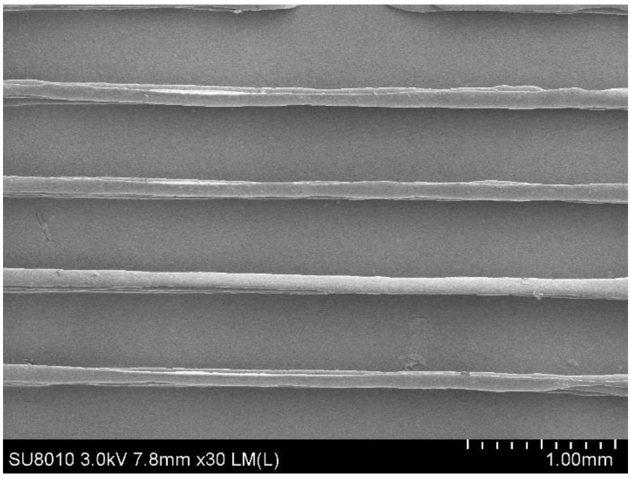

Supplementary Figure 1. SEM of printed stripes on the hydrogel interface.

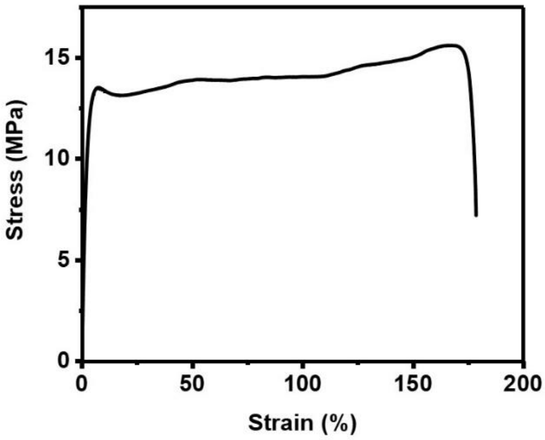

Supplementary Figure 2. Stress-strain curve of FEP.

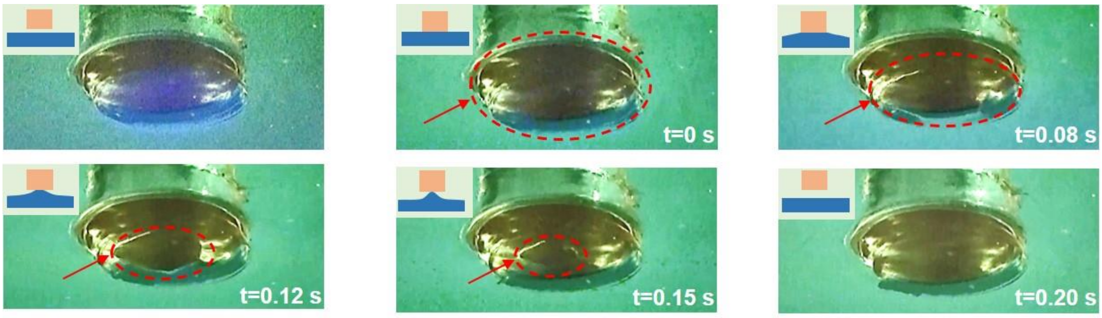

Supplementary Figure 3. Captured pictures of the real-time separation during printing a cylinder with a 20 mm diameter using the HG5-4mm hydrogel as the separation interface.

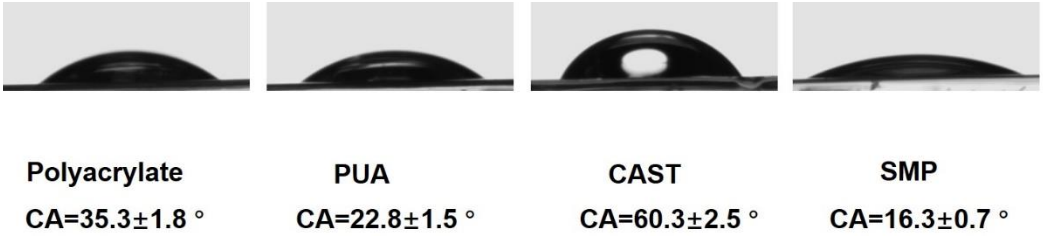

Supplementary Figure 4. Contact angles of different resins on the hydrogel interface

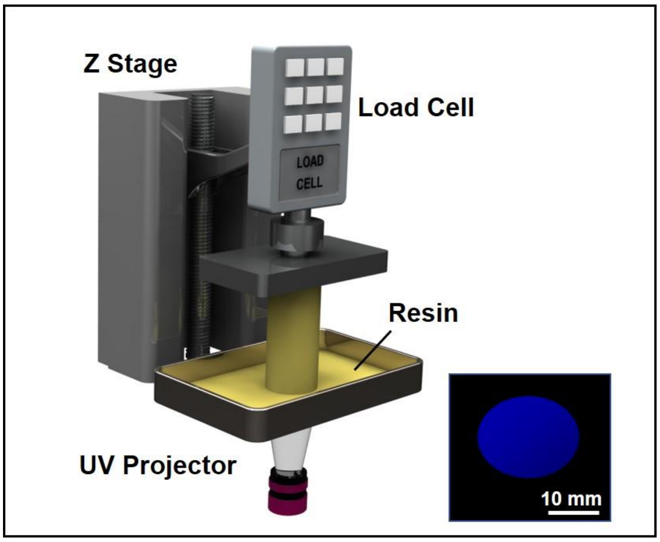

Supplementary Figure 5. Scheme of the bottom-up DLP printer mounted with a load cell for measuring the real-time separation force.

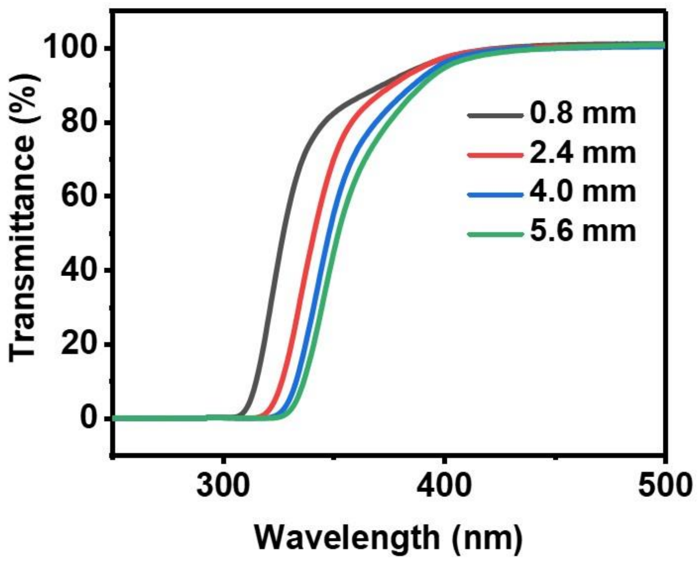

Supplementary Figure 6. Transparence of the curing window: glassy tank + hydrogel layer with different thickness.

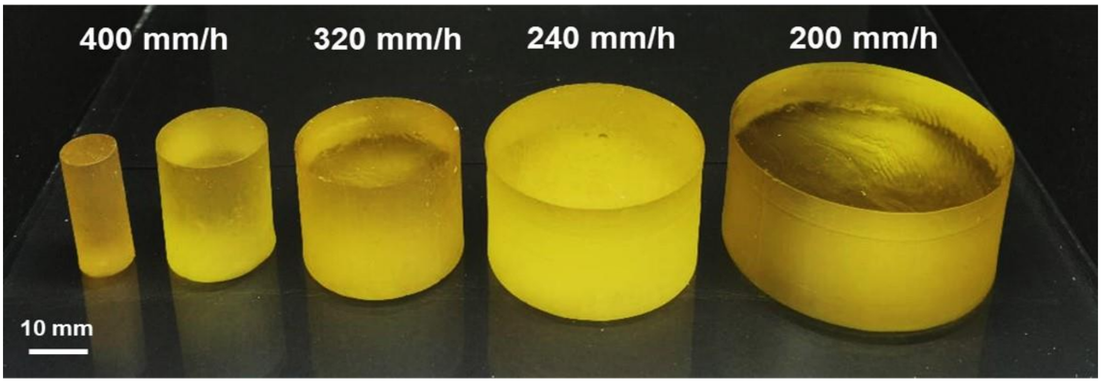

Supplementary Figure 7. Optical pictures of different cylinders printed with different printing speed.

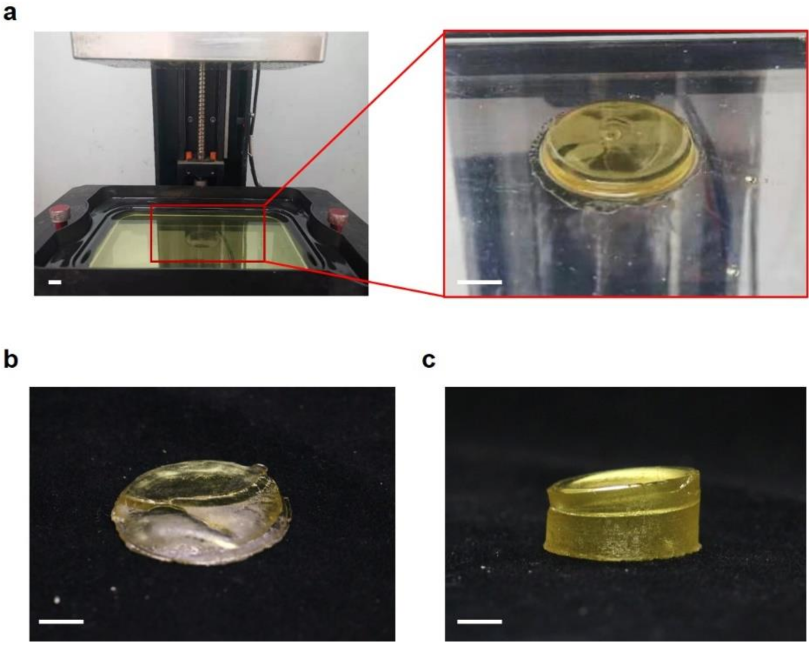

Supplementary Figure 8. Printing failure when FEP is applied. (a) sticky of the printed objects to the FEP. (b) and (c) local defects.

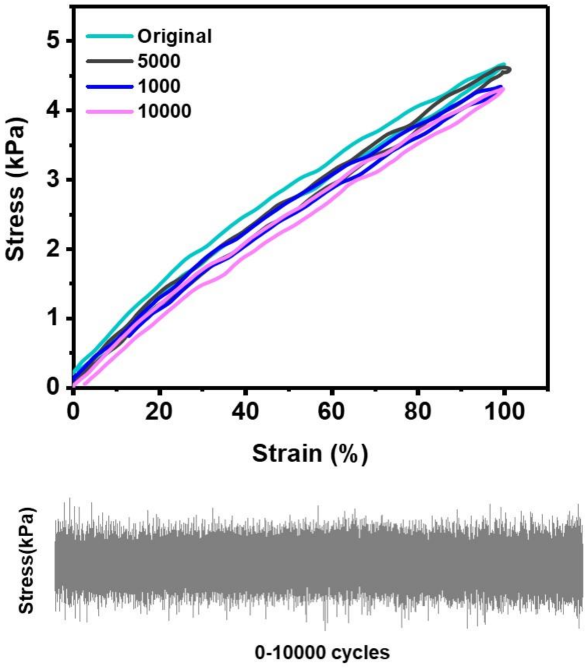

Supplementary Figure 9. (up) Stress-strain curves of HG5 hydrogels undergoing 1000, 5000,10000 cycles consecutive stretch. (down) records of the stress during 10000 cycles consecutive stretch.

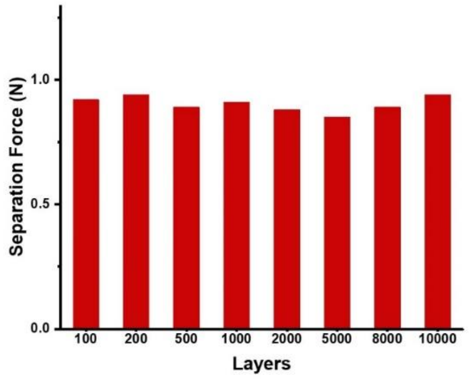

Supplementary Figure 10. Real time separation force during ten thousand consecutive separation.

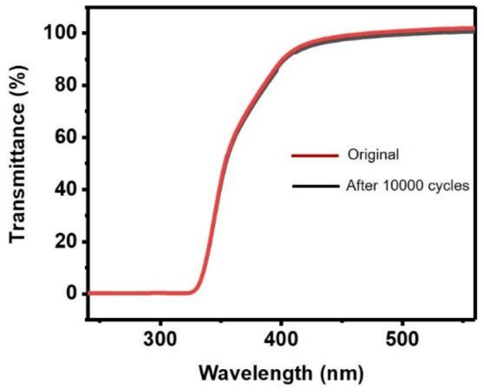

Supplementary Figure 11. Transparency of the hydrogel interface after ten thousand consecutive separations.

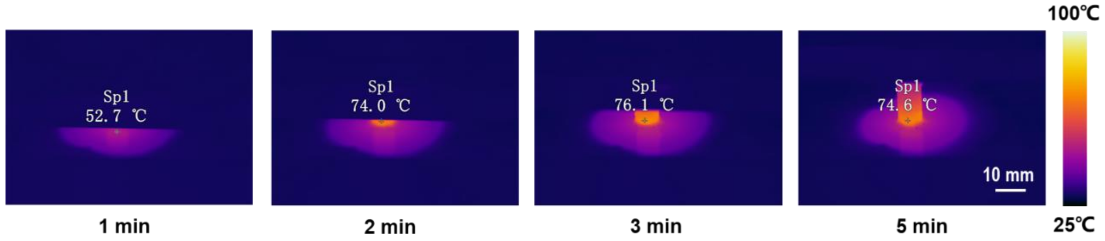

Supplementary Figure 12. Infrared information during the rapid printing (400 mm/h).

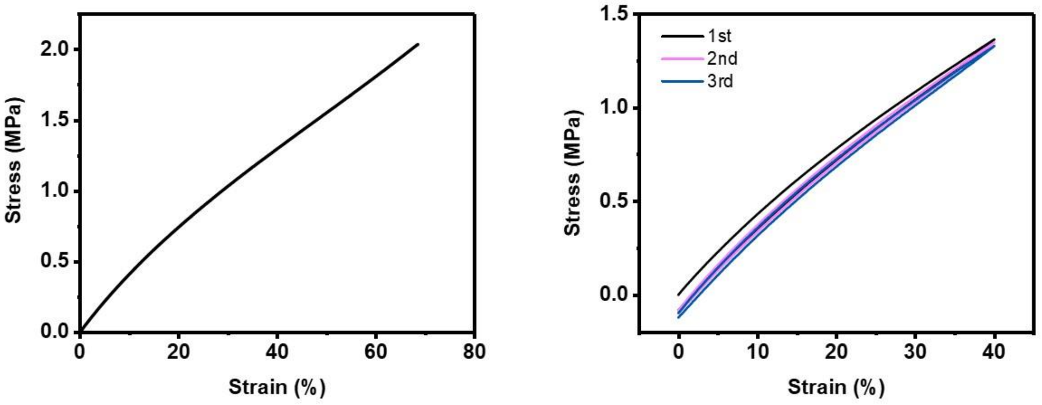

Supplementary Figure 13. (a) Stress-strain curve of the elastomer. (b) The tensile loading-unloading cycle test for the elastomer.

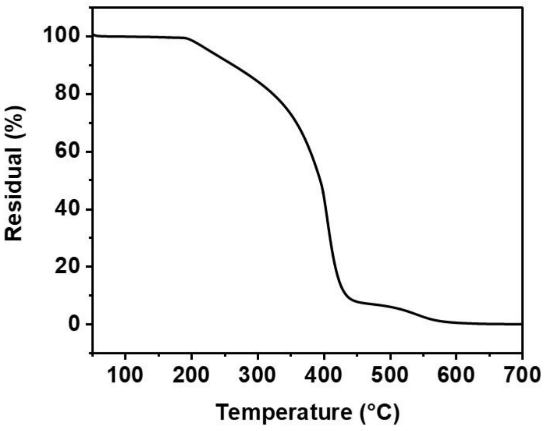

Supplementary Figure 14. Thermogravimetric curve of the casting resin.

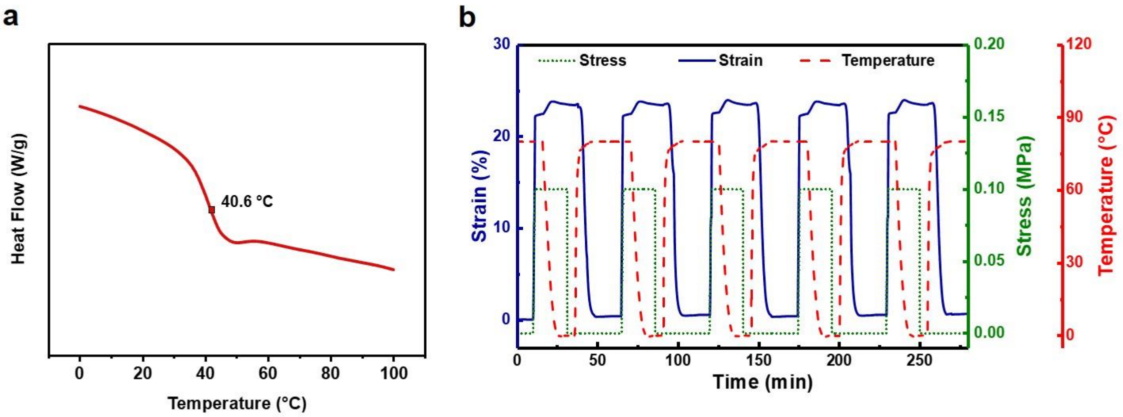

Supplementary Figure 15. (a) DSC curve of the SMP resin. (b) The consecutive shape memory cycles.
