# Supplementary material for: Rapid digital light 3D printing enabled by a soft and deformable hydrogel separation interface
Source: Nat Commun. 2021 Oct 18;12:6070. doi: 10.1038/s41467-021-26386-6 (PMC8523520; doi:10.1038/s41467-021-26386-6)
Supplement: Supplementary file 2 — Description of Additional Supplementary Files [file 41467_2021_26386_MOESM2_ESM.pdf]

Description of Additional Supplementary Files

File Name: Supplementary Movie 1
Description: Real time separation process.

File Name: Supplementary Movie 2
Description: 3D printing of a solid squirrel model.

File Name: Supplementary Movie 3
Description: 3D printing of a lattice model.
